# Application of thermal imaging combined with machine learning for detecting the deterioration of the cassava root

**DOI:** 10.1016/j.heliyon.2023.e20559

**Published:** 2023-09-29

**Authors:** Jetsada Posom, Chutatip Duangpila, Khwantri Saengprachatanarug, Seree Wongpichet, Jiraporn Onmankhong

**Affiliations:** a Department of Agricultural Engineering, Faculty of Engineering, Khon Kaen University, Khon Kaen,40002, Thailand; b Department of Agricultural Engineering, School of Engineering, King Mongkut's Institute of Technology Ladkrabang, Bangkok, 10520, Thailand

**Keywords:** Cassava root, Thermal imaging, Machine learning, Deterioration levels

## Abstract

Freshness is an important parameter that is indexed in the quality assessment of commercial cassava tubers. Cassava tubers that are not fresh have reduced starch content. Therefore, in this study, we aimed to develop a new approach to detect cassava root deterioration levels using thermal imaging with machine learning (ML). An underlying assumption was that nonfresh cassava roots may have fermentation inside that causes a difference in the inner temperature of the tuber. This creates the opportunity for the deterioration level to be measured using thermal imaging. The features (pixel intensity and temperature) that were extracted from the region of interest (ROI) in the form of tuber thermal images were analyzed with ML. Linear discriminant analysis (10.13039/100003090LDA), k-nearest neighbor (kNN), support vector machine (SVM), decision tree, and ensemble classifiers were applied to establish the optimal classification modeling algorithms. The highest accuracy model was developed from thermal images of cassava roots captured in a darkroom under a control temperature of 25 °C in the measurement chamber. The LDA, SVM, and ensemble classifiers gave the best overall performance for the discrimination of cassava root deterioration levels, with an accuracy of 86.7%. Interestingly, under uncontrolled environmental conditions, the combination of thermal imaging plus ML gave results that were of lower accuracy but still acceptable. Thus, our work revealed that thermal imaging coupled with ML was a promising method for the nondestructive evaluation of cassava root deterioration levels.

## 1 Introduction

Cassava (*Manihot esculenta* Crantz) is a woody shrub with edible starch roots (tubers) that is extensively cultivated in many countries in Latin America, Asia, and sub-Saharan Africa [1]. Thailand is the second-largest producer of cassava roots worldwide and the largest exporter of cassava products because of low domestic consumption [2,3]Cassava export volume from Thailand was approximately 30.1 million tons in 2021 [4]. Cassava is the third major economic agricultural product in Thailand after rubber and rice, and cassava is cultivated in 48 of Thailand's 76 provinces [2,4]. Cassava roots contain the nutrients vitamin C, carotenoids, calcium, potassium, iron, magnesium, copper, zinc, and manganese [5]. The roots also have a high starch content, which is the main calorie source for more than 800 million people around the world [6]. Moreover, cassava can be a viable substitute for wheat [7]. It is not only a food source for humans; it also provides nutrition for a variety of animals [8]. In addition, cassava roots can be used to produce bioethanol, which is an important alternative to fossil fuels [6].

As a crop, cassava can withstand long periods of drought and erratic rainfall [9]. However, the cassava root is susceptible to physiological stress after harvest, which is referred to as postharvest physiological deterioration (PPD) [10]. In storage, cassava roots can undergo PPD within 48 h of harvest [11,12]. When PPD occurs, the color of the vascular parenchyma changes to either blue/black or brown [13]. Moreover, Yan et al. (2021) reported that the vascular streaking gradually spread to all the stored cassava roots from 12 to 48 h after harvest, which was a PPD symptom [14]. In addition, Sánchez et al. (2011) noted that the starch in cassava tubers from PPD-susceptible (HMC-1) and tolerant (AM 206-5) clones changed over 14 days of storage under ambient tropical conditions. Starch was analyzed each day. PPD levels differed significantly between the two clones (35% and 8%, respectively, at day 14). The cassava tubers lost weight consistently during storage (approximately 10% in two weeks). Moreover, the starch loss was approximately 1% per day, which could have been the result of consistent increases in the total sugars and respiration of tuber tissue [15]. Furthermore, Idowu and Akindele et al. (1994) observed the qualitative changes in cassava gari and fufu after storage of roots for up to four days at an ambient temperature. They found that the acidity of cassava roots did not change appreciably during storage, but moisture and cyanide content decreased by 3.2% and 7.7%, respectively [16]. Once primary PPD sets in, the stored roots become infected with microorganisms that further degrade the sugars through microbiological PPD, which is characterized by the presence of organic acids [17] PPD is influenced by cassava genotype, moisture content of the stored root, temperature, and type of invading microbes. These factors affect the type and rate of PPD and result in various levels of spoilage [13]. Spoilage affects the market value and consumer acceptance of the product [10]. Moreover, some farmers need to collect cassava roots and then transfer them to the factory, which can take considerable time, during which the deterioration of the roots proceeds. In general, the evaluation of cassava root deterioration requires the roots to be broken down to show the physical properties of cassava root deterioration that the human eye can observe. This method is destructive, time-consuming, and labor intensive. For this reason, a rapid and nondestructive way to classify the deterioration of cassava roots was required.

Recently, infrared thermal imaging techniques have been used to solve problems related to fruit quality [18]. The advantages of infrared thermal imaging are high repeatability, easy operation, fast detection speed, and nondestruction of the target [18,19]. Thermal imaging is a practical method that transforms the radiation emitted by an object into a noninvasive distribution of surface temperatures [20]. The changes that may exist within the structure of the evaluated object cause distinct thermal conduction in the material, which affects the direction of heat flow [21]. This indicates that the object being examined will cool or warm up at a variable ratio depending on its structural qualities (either its internal structure or the existence of flaws) [22]. Moreover, PPD of cassava roots begins at the broken or cut surfaces and subsequently spreads to the adjacent storage parenchyma, and the stored starch undergoes structural changes [23]. Therefore, different structures of cassava roots at different times during the storage period are possible at different surface temperatures. Infrared thermal imaging has been used successfully to detect the quality of several fruits. Mohd Ali et al. (2022) reported on the use of infrared thermal imaging to evaluate the qualities (firmness, pH, total soluble solids, moisture content, and color) of pineapple during storage [18]. The prediction performance of pineapple quality was developed using partial least squares regression, which obtained coefficient of determination (R^2^) values up to 0.94 for all the quality parameters of the pineapple varieties [18]. Naik and Patel (2017) used infrared thermal images to classify the grade of mangoes based on their maturity and size [24]. The results showed that accuracy was 89% [24]. Ding et al. (2017) classified grapes as being fresh, slightly or moderately decayed, or seriously decayed using thermal imaging of volatile alcohols [25]. The results showed that thermal images were the correct classification ratings of 100%, 93.3%, and 90%, respectively [25]. Zeng et al. (2020) used thermal imaging to classify bruises on pears [26]. The best test accuracy of prediction reached 99.3%. Chandel et al. (2018) used an RGB camera coupled with a thermal module to model real-time fruit surface temperature (FST), revealing an R^2^ up to 0.90 with the FST derived from the micro-climate sensor. The imager estimated critical conditions for apple sunburn and demonstrated numerous advantages over other techniques [26]. Kuzy et al. (2018) develop a pulsed thermographic imaging system to detect bruised blueberries. They used LDA and SVM algorithms to classify bruise detection on blueberries. Both algorithms had a strong classification performance. Accuracies of up to 88% and 79% were obtained for Farthing and Meadowlark berries, respectively [27]. In addition, Raza et al. (2015) developed a method for identifying fungal contamination in tomatoes with thermal imaging and the SVM algorithm, which had a high accuracy rate of more than 90% [28]. Moreover, Rahman et al. (2021) reported that the ensemble model was utilized for fruit identification from seed images [29]. Currently, algorithms are used in various fields, such as pattern recognition, disease diagnosis, and fraud detection [30]. Furthermore, algorithms find applications in the field of smart agriculture. In this study, the algorithms of LDA, SVM, kNN, decision tree, and ensemble classifiers were applied for detecting the deterioration of cassava roots using thermal image data. LDA is suitable for linearly separated data. LDA is to find the linear combinations of noted attributes that perfectly separate or characterize two or more classes of events or objects [31]. The practical appeal of LDA can be explained by its intrinsically low model complexity and its ability to capture the essential characteristics of the data distributions (mean and covariance) from finite training data. It then estimates the decision boundary using these 'global' characteristics of the data [32]. LDA has found numerous recent applications in the field of agricultural produce inspection, such as identifying grape leaf health [33], classifying apples based on skin color [34] and so on. As a popular machine learning algorithm, the SVM approach emphasizes the concept of maximizing the margin (degree of separation) in the training data. SVM can also be viewed as a discriminative classifier; however, it uses a local separation index (i.e., margin). The SVM decision boundary depends on a subset of the data points (or support vectors) that are close to the decision boundary [32]. The main advantage of SVM is its ability to handle a wide variety of classification problems, including those with high dimensions and non-linearly separable data. SVM is typically used primarily for classifying different types of patterns [35]. This algorithm has been widely used in many fields, such as information retrieval and agriculture, for crop and soil classifications [36]. kNN identifies the nearest neighbors of the testing data for a given input and predicts the category based on certain distance functions [37]. One of the main advantages of the kNN technique is its effectiveness with large training data and its robustness to noisy training data [38]. Several studies have been conducted on the application of data mining techniques to agricultural datasets [39]. In agriculture, the kNN algorithm has been found to be highly effective for the classification of various grains and grain cultivars [40]. Additionally, kNN is utilized in agriculture for the management of machinery, crops, soil, and livestock [40]. Furthermore, decision tree algorithms are the most commonly used methods in classification [41]. The goal of decision trees is to create a model that predicts the value of a target variable by learning simple decision rules inferred from the data characteristics [42]. Decision trees offer an easily understandable modeling technique and simplify the classification process [43]. This technique is capable of handling both complete and incomplete data and is applied to classification problems with various agricultural datasets [40]. Ensemble classifiers in machine learning achieve this objective by combining multiple simple base models (decision trees, kNN, and others) to create a powerful model that yields improved predictive outcomes [44]. Ensemble classifiers are suited to complex and high-dimensional datasets as well as datasets with noisy or incomplete features. In recent times, the ensemble classifier algorithm has been used for the classification and identification of multiple crops in an agricultural environment and for general image classification [45]. The five algorithms mentioned above are the same as those employed in supervised learning [38,46]. However, the five algorithms mentioned above differ in principle, methodology and performance characteristics. Each has its own strengths and weaknesses, which depend on the specific characteristics of the data problem. Moreover, all five algorithms mentioned are applied in agricultural fields for classification. Therefore, it is possible that all five algorithms are predict casava roots using thermal imaging data. In this study, all five algorithms were applied to compare the optimal accuracy for detecting the deterioration of cassava roots. Moreover, environmental conditions have been reported to affect thermal imaging. Yogesh et al. (2018) compared the approaches of various segmentation methods for identification of defected regions in apples. The results showed that texture-based segmentation perceived better results than that of shape, size, and color. They also indicated that the drawback is that the thermal behavior of fruit is dependent on environmental factors [47]. Moreover, Xu and Ying (2004) suggested that thermal imaging has the potential for citrus detection. They did not report the temporal temperature variation, the effect of sunlight, and other environmental conditions that could influence thermal imaging [48]. In addition, Melesse et al. (2022) suggest that the detection of thermal images works well in all brightness levels to distinguish the targets easily from the background, depending on the radiation difference [49]. Therefore, environmental conditions, including the brightness level and temperature during the experiment were also of interest in this research. However, to date no such method has been used to measure the deterioration of cassava roots.

Therefore, the objectives of this study were to (1) study the feasibility of detecting deteriorated cassava levels using infrared thermal imaging, (2) compare the performance of the classification algorithm, including LDA, SVM, kNN, decision tree and ensemble classifiers, (3) compare the ability of infrared thermal imaging cameras in bright and darkrooms for detecting deteriorated cassava roots, and (4) inspect and compare the ability of the thermal imaging camera to detect root deterioration in the control room at 25 °C and at ambient temperatures.

## 2 Materials and methods

### 2.1 Cassava root samples

Fresh cassava root samples of 39 tubers were collected randomly from storage piles at the Thai Wah Public Company Limited in Tha Khantho District, Kalasin Province, Thailand. The cassava tubers were cleaned without using water, and then sand and soil were swept away. These tubers were freshly harvested (within 24 h), and the lengths of the tubers were between 25 and 30 cm. The cassava variety was Kasetsart 50, which is the most important cassava cultivar in Thailand and probably in the world.

### 2.2 Thermal image collection

**Fig. 1:**
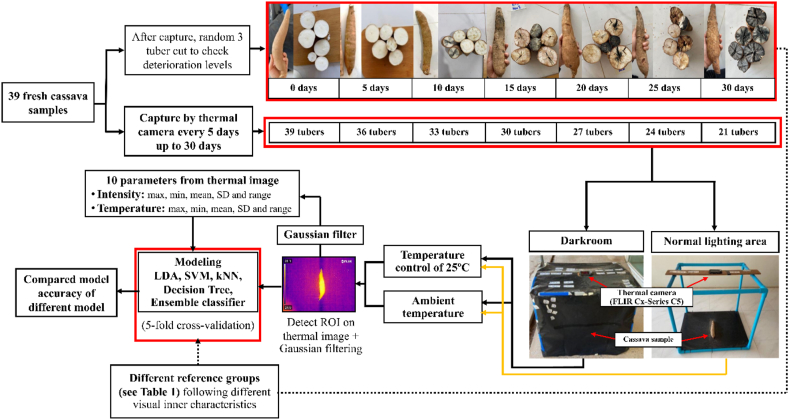
Schematics of the experiment.

The measurement system consisted of a measurement chamber (1 cm long × 1 cm width × 1 cm height), and a thermal imaging camera (FLIR Cx-Series C5, FLIR Systems, Wilsonville, Oregon, USA) was used in this research. This camera's spectra and temperature ranged from 8 to 14 μm and −20 to 400 °C, respectively, with a display resolution of 640 × 480 pixels. Temperature accuracy: 0–100 °C (32–212 °F): ±3 °C (±5.5 °F) and 100–400 °C (212–752 °F): ±3%. Ambient temperature of 15–35 °C ± 3 °C (59–95 °F ± 5.5 °F) and object temperature above 0 °C (32 °F) can be detected, as shown in Fig. 1.

Fig. 1 shows a flow diagram of the experiment. The thermal camera was installed at the middle top of the measurement chamber. An emissivity coefficient for cassava tubers of 0.67 was set [50]. A blackbody reference source made from a black sheet (rigid PVC) that could absorb and radiate electromagnetic radiation was used as the background for calibrating the thermal imaging device. The distance between the samples and the camera was fixed at 80 cm, which enabled a clear image.

Thermal images of the cassava tuber samples were captured every five days after storage from day 0 (fresh) through to 30 days of incubation. Initially, the number of tubers was 39. At day 0, 39 tubers were captured; after that, three tubers were randomly cut in cross sections in order to check their inner properties by visual inspection. The remaining 36 tubers were stored for five days and then were captured. Three tubers were randomly collected and cut in cross sections for visual inspection of their inner properties. Then, the remaining 33 tubers were stored for five days, and then captured, and three collected tubers were cut in cross sections again. The remaining samples are subjected to the same experiment process mentioned earlier from storage to 30 days after storage. Therefore, after 30 days, 21 tubers remained.

To evaluate the performance of the model created using different photographic environments, testing of different treatments was conducted. The tuber samples were captured under different conditions, as follows.

1. Dark conditions and a control temperature of 25 ± 2 °C
2. Dark conditions and an ambient temperature of 27 ± 5 °C
3. Normal light and a control temperature of 25 ± 2 °C
4. Normal light and an ambient temperature of 27 ± 5 °C

For the dark conditions, the sample was captured in a measurement chamber that was covered by a plastic black sheet to block the light.

For the control temperature, the experiment was done in a room with a control temperature of 25 ± 2 °C. Before the experiment, the sample was placed in the control temperature of 25 ± 2 °C room for 2 h before capture.

For the normal light conditions, the sample was captured in a room with the light turned on and captured in a measurement chamber not covered by a black sheet.

For the ambient temperature conditions, the experiment was conducted in room with an uncontrolled temperature.

Therefore, each tuber underwent four treatments, and each treatment had 210 images per model.

### 2.3 Thermal image processing

The thermal images of cassava samples were analyzed using MATLAB software (Version R2022a, The MathWorks, Natick, MA, US). The thermal image processing is shown in Fig. 1. The thermal images of the cassava tuber samples were converted to grayscale images. The mean pixel value of each thermal image was determined by selecting a region of interest (ROI) to eliminate image noise. The ROI was segmented using an image segmentation technique in which an optimal threshold value was calculated to separate the background from the thermal image. After extracting the ROI, the thermal image was converted to a binary image. In order to enhance the quality of the image, the binary image was then converted to a grayscale image using a Gaussian filter. The feature extraction from the thermal images was obtained from the segmented images based on the pixel values known as thermal parameters.

To determine a correlation between deterioration levels and the thermal parameters of the cassava tubers, a calculation of feature values was determined based on the pixel values. Using the features extracted from image regions, 10 features were used based on the pixel value measurement. The selected thermal parameters of ROI included intensity and temperature: maximum, minimum, average, standard deviation, and range, which were obtained from the pixel values extracted from the ROI. Therefore, there were 10 features used for model development. All the thermal parameters were derived from the remarkable parts of the thermal image as compact feature vectors.

### 2.4 Classification model

For model development, the 10 thermal parameters, including maximum, minimum, average, standard deviation, range of ROI intensity, and temperature coupled with deterioration levels of cassava tubers were used to develop the classification model.

The deterioration of cassava samples was divided into three groups: fresh cassava (no deterioration) (L0), moderate deterioration (L1), and high deterioration (L2). The characteristics of the L0, L1, and L2 groups were that they were pure white, starting to develop black dots with cracks, and becoming black, respectively (Table 1). For the reference samples, the sample was cut in cross sections immediately after capturing to observe the deterioration level. To study the most suitable deterioration levels classified by thermal imaging, the deterioration levels of cassava tubers were divided into four models (see Table 1) based on the days after harvesting, as follows.

Model 1 L0 (0 days), L1 (5–20 days), and L2 (25–30 days)

Model 2 L0 (0–5 days), L1 (10–20 days), and L2 (25–30 days)

Model 3 L0 (0–5 days), L1 (10–15 days), and L2 (20–30 days)

Model 4 L0 (0 days), L1 (5–15 days), and L2 (20–30 days)

The performance of the four models was compared to find the appropriate model to classify deterioration levels of cassava tubers.

The classification models were developed using LDA, SVM, kNN, decision tree, and ensemble classifiers, and validated using 5-fold cross-validation sets. The classification models were developed using the Classification Learner app in the Statistics and Machine Learning Toolbox of MATLAB. Data analysis was developed using the MATLAB software (Version R2022a, The MathWorks, Natick, MA, US). The results of five algorithms were compared, and the highest accuracy was found for the appropriate classification model of cassava deterioration.

**Table 1:** Assigning the days after harvesting as different deteriorated levels.

	Days after harvesting						
							
**Number of captured tubers**	39 (start 0 day)	36 (39-3 tubers)	33 (36-3 tubers)	30 (33-3 tubers)	27 (30-27 tubers)	24 (27-3 tubers)	21 (24-3 tubers)
**Model**	0 days	5 days	10 days	15 days	20 days	25 days	30 days
Model 1	L0 (39 s)	L1 (126 s)				L2 (45 s)	
Model 2	L0 (75 s)		L1 (90 s)			L2 (45 s)	
Model 3	L0 (75 s)		L1 (63 s)		L2 (72 s)		
Model 4	L0 (39 s)	L1 (99 s)			L2 (72 s)		

s: samples.

L0: Fresh cassava (no deterioration).

L1: Moderate deterioration.

L2: High level of deterioration.

## 3 Results and discussion

### 3.1 Deterioration levels of cassava samples

The quality attributes of cassava were the three different deterioration levels after harvesting. Four models based on different days of fermentation were obtained for all the quality attributes of cassava (L0 to L2). To find an appropriate classification level, each model was divided into deterioration levels based on days after harvesting, as described in Table 1. In this study, the cassava tubers were pure white after harvesting (0 days), and showed no deterioration. The cassava tubers started to develop black dots after five days of storage, and after 10 days, they were cracked. Finally, the tubers became black, and cracks had spread to the whole sample after 30 days of storage. Most of the cassava tubers deteriorated within 3–4 days after harvest, which was consistent with the observations of Luna et al. (2021) [51] and [52].

### 3.2 Changes in thermal parameter

**Fig. 2:**
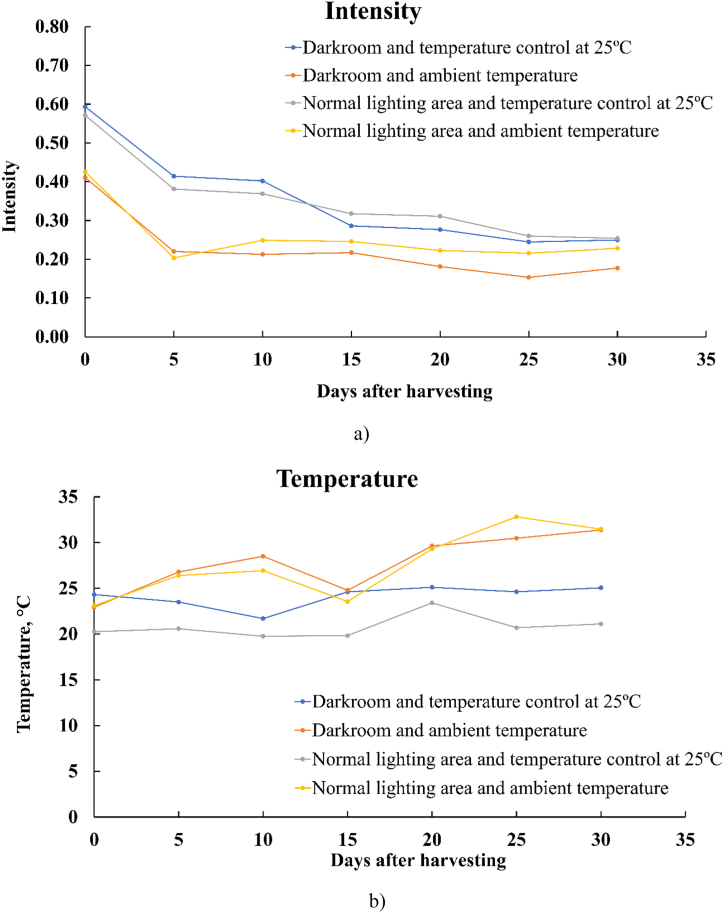
Averaged ROI of different thermal image capture situations: a) intensity and b) temperature.

The thermal parameters were chosen based on the feature extraction of cassava thermal images to develop the classification model for the detection of deterioration levels of the cassava. The pixel intensity and temperature of thermal parameters on different days after harvest following the thermal image capture situation are presented in Table 2. In this study, ANOVA was performed to analyze the effect of days after harvest and thermal image capture situations on the intensity and temperature of thermal parameters. Both thermal parameters were influenced by the number days after harvest and the thermal image capture situations. The difference between the days after harvest showed a significant (*p* < 0.05) influence on the intensity parameter. The intensity parameter decreased with an increase in the number of days after harvest (Fig. 2a). In contrast, the temperature parameter increased with an increase in the number of days after harvest (*p* < 0.05) (Fig. 2b). However, the intensity and temperature parameters did not significantly differ between 20, 25, and 30 days after harvest. Therefore, the results indicated that the pixel intensity and temperature parameters were different depending on the number of days after harvesting. This indicated that it was possible to apply the intensity and temperature thermal parameters to classify the deterioration levels of cassava. Changes in the intensity and temperature parameters might have been caused by PPD, which was influenced by incubation in this study, resulting in deterioration. The internal characteristics of the stored cassava storage were a blue/black or brown color [13]. In storage, cassava becomes infected with microorganisms that further degrade the sugars through microbiological PPD, which is characterized by the presence of organic acids [53]. In the same way, Baranowski et al. (2008) used thermography to diagnose watercore in apples. The temperature parameter of apples was used to classify them as being diseased or healthy. The initial stages of temperature for watercored apples were significantly lower than those for healthy apples. The results showed a good correlation between the temperature and density of the fruit tissue [54]. In other research, Van Zeebroeck et al. (2007) reported that apple harvest time had an effect on apple bruising [55].

**Table 2:** Thermal parameters based on cassava feature extraction at different days after harvesting. The thermal parameters used are defined as thermal parameters based on cassava feature extraction at different deterioration levels.

	Intensity					Temperature, °C				
Days	Darkroom and temperature control at 25 °C	Darkroom and ambient temperature	Normal lighting area and temperature control at 25 °C	Normal lighting area and ambient temperature	AVE	Darkroom and temperature control at 25 °C	Darkroom and ambient temperature	Normal lighting area and temperature control at 25 °C	Normal lighting area and ambient temperature	AVE
0	0.59	0.41	0.57	0.43	0.50^a^±0.10	24.30	22.90	20.30	23.10	22.70^b^ ± 1.70
5	0.41	0.22	0.38	0.20	0.30^b^ ± 0.11	23.50	26.80	20.60	26.40	24.30^ab^ ± 2.90
10	0.40	0.21	0.37	0.25	0.31^b^ ± 0.09	21.70	28.50	19.80	26.90	24.20^ab^ ± 4.20
15	0.29	0.22	0.32	0.25	0.27^bc^±0.04	24.60	24.80	19.80	23.60	23.20^b^ ± 2.30
20	0.28	0.18	0.31	0.22	0.25^c^±0.06	25.10	29.60	23.40	29.30	26.90^a^±3.10
25	0.26	0.15	0.26	0.22	0.22^c^±0.05	24.60	30.50	20.70	32.80	27.10^a^±5.50
30	0.25	0.18	0.25	0.23	0.23^c^±0.04	25.10	31.40	21.10	31.50	27.20^a^±5.10
AVE	0.35^a^±0.13	0.23^b^ ± 0.09	0.35^a^±0.11	0.26^b^ ± 0.08		24.10^b^ ± 1.20	27.80^a^±3.10	20.80^c^±1.20	27.70^a^±3.70

Fig. 2 shows the average ROI of different thermal image capture situations. For the thermal image capture situation, the darkroom and normal lighting area with temperature control at 25 °C were not significantly different in intensity. Moreover, the darkroom and normal lighting areas at ambient temperature did not differ significantly in intensity. It was recognized that the thermal image capture situation may be influenced by varying temperature. Therefore, to carry out the thermal image measurements on cassava, it was necessary to define stable conditions (humidity and temperature) for capturing the thermal images throughout the experimentation.

### 3.3 Classification models

In this study, five multivariate algorithms were tested to determine the most suitable for the classification of the deterioration levels of cassava. The LDA, SVM, kNN, decision tree, and ensemble classifier models were applied to classify the deterioration level based on the intensity and temperature thermal parameters. The average classification accuracies of cassava tubers at different deterioration levels are displayed in Table 3.

**Table 3:** The average classification accuracies of cassava tubers at different deterioration levels using various multivariate algorithms.

	Correct classification (%)				
Model	LDA	SVM	kNN	Decision Tree	Ensemble classifier
Darkroom and temperature control at 25 °C					
Model 1	79.5	83.8	80	77.1	82.4
Model 2	62.9	62.9	68.1	64.8	63.8
Model 3	71	71.4	73.3	69.5	71.0
Model 4	**86.7**	**86.7**	86.2	83.8	**86.7**
Darkroom and ambient temperature					
Model 1	75.7	78.1	77.1	72.9	78.6
Model 2	56.7	67.6	63.3	61.1	69.5
Model 3	58.1	61.9	61.4	61.1	63.8
Model 4	71.9	72.4	71.4	71.4	74.3
Normal lighting area and temperature control at 25 °C					
Model 1	81	85.7	81.9	81	83.3
Model 2	69	71.4	74.3	65.2	71.4
Model 3	63.3	69.5	73.3	64.3	68.1
Model 4	73.8	75.2	78.1	74.8	79.5
Normal lighting area and ambient temperature					
Model 1	82.9	86.2	85.2	86.2	86.2
Model 2	71.9	79	76.7	75.2	81.9
Model 3	65.7	74.8	70	62.9	73.8
Model 4	76.7	81.9	77.6	73.3	80.5
Darkroom + Normal lighting area with temperature control at 25 °C					
Model 1	81	82.6	81.4	77.9	82.9
Model 2	67.9	67.9	68.3	61.4	66.9
Model 3	71.9	72.1	76.0	64.8	72.9
Model 4	78.6	82.4	83.1	78.3	82.4
Darkroom + Normal lighting area with ambient temperature					
Model 1	73.1	86.2	81.0	79.3	81.2
Model 2	60.7	72.9	67.6	68.1	72.4
Model 3	55.2	67.1	64.0	62.4	70.2
Model 4	72.4	76.9	71	71.0	74.3
Total data					
Model 1	72.4	79.9	76.7	72.3	77.7
Model 2	57.1	68.1	65.4	59.3	67.3
Model 3	61.4	67.3	68.1	62.9	67.1
Model 4	70.7	76.5	75	69.6	76.8

Multivariate algorithms used: LDA = linear discriminant analysis, kNN = k-nearest neighbor, SVM = support vector machine.

**Fig. 3:**
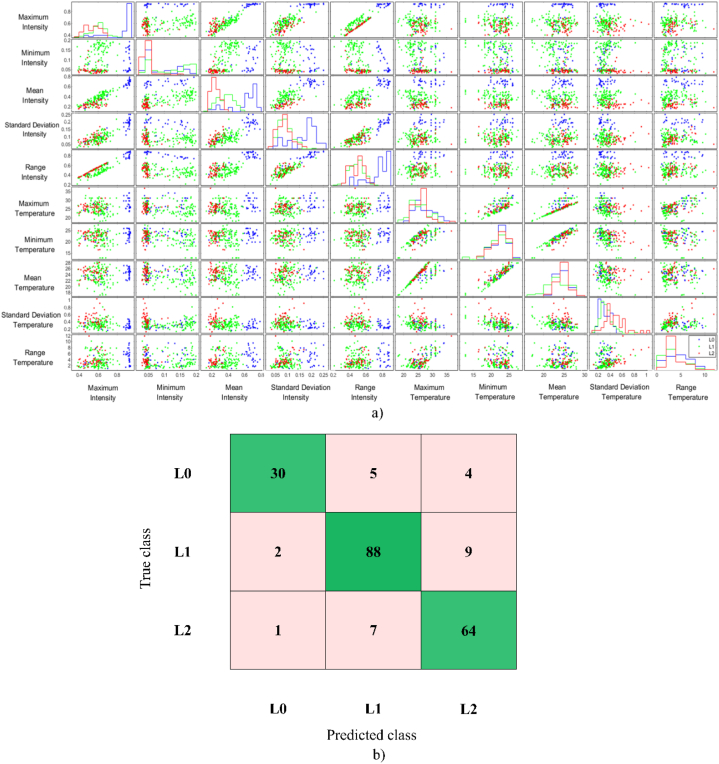

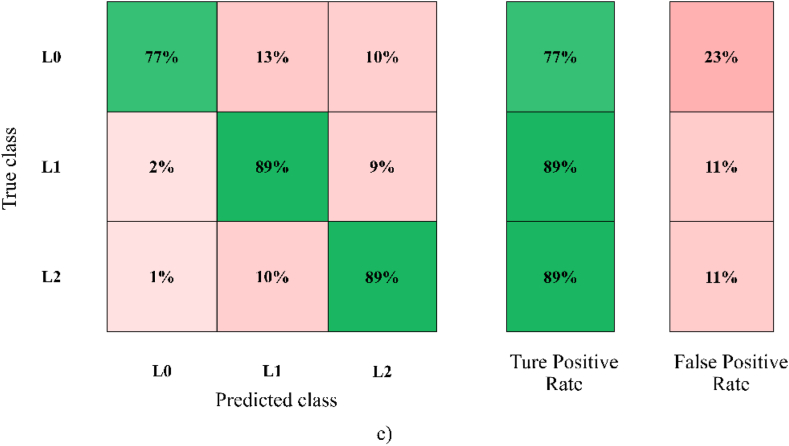
A) visualizing multivariate data in scatter plot with three groups PPD based on model 4, b) number of observations, and c) confusion matrix of LDA-model 4 from darkroom and temperature control at 25 °C.

In the comparison of the four different thermal image capture situations, the combination of the darkroom and temperature control at 25 °C showed the best overall classification performance (86.7%), developed with LDA, SVM, and the ensemble classifier models with Model 4. Those algorithms had the same accuracy. Fig. 3a shows the LDA scatter plot that was applied to define the clustering capability at the three different deterioration levels (L0, L1, and L2) based on the thermal parameters following Model 4. The LDA scatter plot shows that L0 was distinctly separated from L1 and L2. Moreover, L1 had a small amount of overlap with L2. This may have been because some samples of L1 deteriorated more quickly than most samples due to the physical and chemical properties of the cassava sample. However, it was only a small part of the deterioration level groups where the scatter plot overlapped. Therefore, the intensity and temperature parameters were important in the classification of the deterioration levels of cassava used to describe the effects of postharvest deterioration. The LDA results showed that the thermal parameters could be used to classify cassava according to the deterioration levels, including L0, L1, and L2. Fig. 3b and c shows the confusion matrix of three levels of the deterioration results based on 5-fold cross-validation by the LDA methods with Model 4 with darkroom and temperature control at 25 °C. The actual deterioration levels of classification are provided in each row, while the prediction levels are exhibited in each column. Also shown is the number of observations and percentage of classification accuracy (true positive and negative rates). The average accuracy was 86.7% (L0: 77%, L1: 89%, L2: 89%). All the classification accuracies in this study showed that thermal imaging could feasibly be used to classify deterioration levels in cassava root tubers. However, the thermal image capture situations influenced the classification accuracy. The best thermal image capture situations were in a darkroom, in which light probably did not interfere with the capture of the thermal image. Performing the imaging in the dark eliminated noise interference. In addition, the temperature being controlled at 25 °C resulted in the best thermal image capture. The results showed that a controlled temperature was more effective than an ambient temperature. Moreover, the defining of the reference groups (L0, L1, and L2) of deteriorated cassava after harvest had an influence on the prediction of the level of deterioration. In this study, the method of separation of the reference groups in Model 4 provided helpful reference groups for deterioration determination. Model 4 was divided into three reference groups consisting of L0 (0 days), L1 (5, 10, and 15 days), and L2 (20, 25, and 30 days), which were related to the statistical results. In Model 4, L0 was assigned to immediate postharvest groups (0 days), in which there was no deterioration. It is possible that the physical and chemical properties of the cassava tubers remained unchanged, resulting in similar thermal parameters. The L0 group of Model 1 was also defined in the same way as Model 4, which had a higher accuracy than Models 2 and 3. In the same way, the L1 and L2 groups of Model 4 were separated from Model 1. However, the best reference group was Model 4. It might have been that each level had quite similar physical and chemical characteristics. Therefore, the accuracy of the LDA, SVM, and ensemble classifier algorithm models with Model 4 were high.

In this study, the best results were with the LDA, SVM, and ensemble classifier algorithms with Model 4, which were able to classify the deterioration level of cassava with high accuracy. LDA and SVM are both linear classifiers. They predict by finding a linear decision boundary that separates the different deterioration levels. This can be very effective for data that is linearly separable, and it can also be more robust to noise than nonlinear classifiers like kNN and decision tree. Ensemble classifiers are multiple-base classifiers, and they combine the predictions of these base classifiers to make a final decision. This can help to reduce the variance of the predictions, and it can also make the classifier more robust to overfitting. The high accuracy depends on the specific data set and the problem that is being solved. In this study, the data was suitable for LDA, SVM, and ensemble classifier algorithms for classifying deterioration levels. Until now, the conventional methods of cassava tuber deterioration classification were destructive. However, our new method overcomes this problem. Thermal imaging with multivariate algorithms such as LDA, SVM, and ensemble classifier can be used to accurately assess the levels of deterioration of cassava tubers in a quick, cost-effective, and nondestructive fashion.

## 4 Conclusion

This study demonstrates the feasibility of using thermal imaging to evaluate the deterioration levels of cassava at three different levels. The thermal parameters obtained from the feature extraction method of intensity and temperature included average, minimum, maximum, standard deviation, and range, and these were utilized to distinguish fresh cassava (no deterioration), moderate deterioration, and high level of deterioration. The LDA, SVM, kNN, decision tree and ensemble classifier models were applied to differentiate cassava tubers into three deterioration level groups. By comparing the performance of the five multivariate tools, the highest performance was obtained with the LDA, SVM, and ensemble classifier algorithms and Model 4, with an overall accuracy of 86.7% for cassava deterioration detection. These classification models provided better performance in darkroom conditions than in bright rooms for detecting deteriorated cassava roots. Moreover, the temperature control at 25 °C was found to be a more suitable condition than ambient temperature for comparing the thermal imaging camera's ability to detect root degradation. These classification models were a darkroom and temperature control at 25 °C. These models described the configuration of a thermal imaging system, which provided a realistic scenario for the detection of cassava deterioration levels. The development of thermal imaging coupled with multivariate analysis contributes to the advance of noninvasive techniques for the quality evaluation of cassava. Future studies should focus on validating and improving the performance model by increasing the number of samples and field locations and the size of samples for the input of thermal image data.

## Author contribution statement

Jetsada Posom; Jiraporn Onmankhong: Conceived and designed the experiments; Performed the experiments; Analyzed and interpreted the data; Wrote the paper. </p>

Chutatip Duangpila: Conceived and designed the experiments; Performed the experiments. </p>;

Khwantri Saengprachatanarug; Seree Wongpichet: Contributed reagents, materials, analysis tools or data. </p>

## Additional information

No additional information is available for this paper.

## Declaration of competing interest

The authors declare that they have no known competing financial interests or personal relationships that could have appeared to influence the work reported in this paper.

## References

[1] Padi R.K., Chimphango A., Roskilly A.P. (2022). Economic and environmental analysis of waste-based bioenergy integration into industrial cassava starch processes in Africa. Sustain. Prod. Consum..

[2] Buddhakulsomsiri J., Parthanadee P., Pannakkong W. (2018). Prediction models of starch content in fresh cassava roots for a tapioca starch manufacturer in Thailand. Comput. Electron. Agric..

[3] Prasara-A J., Gheewala S.H. (2021). An assessment of social sustainability of sugarcane and cassava cultivation in Thailand. Sustain. Prod. Consum..

[4] Manakitsomboon H. (2022). https://www.statista.com/statistics/1041126/thailand-cassava-production-volume/#statisticContainer.

[5] Tappiban P., Smith D.R., Triwitayakorn K., Bao J. (2019). Recent understanding of starch biosynthesis in cassava for quality improvement: a review. Trends Food Sci. Technol..

[6] Huang T., Luo X., Fan Z., Yang Y., Wan W. (2021). Genome-wide identification and analysis of the sucrose synthase gene family in cassava (Manihot esculenta Crantz). Gene.

[7] Odey G.N., Lee W.Y. (2020). Evaluation of the quality characteristics of flour and pasta from fermented cassava roots. Int. J. Food Sci. Technol..

[8] Kayode B.I., Kayode R.M.O., Salami K.O., Obilana A.O., George T.T., Dudu O.E., Adebo O.A., Njobeh P.B., Diarra S.S., Oyeyinka S.A. (2021). Morphology and physicochemical properties of starch isolated from frozen cassava root. LWT.

[9] Enesi R.O., Hauser S., Pypers P., Kreye C., Tariku M., Six J. (2022). Understanding changes in cassava root dry matter yield by different planting dates, crop ages at harvest, fertilizer application and varieties. Eur. J. Agron..

[10] Oduor L.A., Owino W., Ateka E.M., Ambuko J. (2017). Effect of surface coatings on the shelf life and quality of cassava. J. Food Res..

[11] Wheatley C., Lozano C., Gomez G., Cock, J.H, Reyes, J.A (1985). Cassava: Research, Production and Utilization.

[12] Wu X., Xu J., Ma Q., Ahmed S., Lu X., Ling E., Zhang P. (2022). Lysozyme inhibits postharvest physiological deterioration of cassava. J. Integr. Plant Biol..

[13] Mbinda W., Mukami A. (2022). Breeding for postharvest physiological deterioration in cassava: problems and strategies. CABI Agriculture and Bioscience.

[14] Yan Y., Zhao S., Ding Z., Tie W., Hu W. (2021). Comparative transcriptomic analysis of storage roots in cassava during postharvest physiological deterioration. Plant Mol Biol Report.

[15] Sánchez T., Dufour D., Moreno J.L., Pizarro M., Aragón I.J., Domínguez M., Ceballos H. (2013). Changes in extended shelf life of cassava roots during storage in ambient conditions. Postharvest Biol. Technol..

[16] Idowu M.A., Akindele S.A. (1994). Effect of storage of cassava roots on the chemical composition and sensory qualities of gari and fufu. Food Chem..

[17] Uarrota V.G., Moresco R., Coelho B., Nunes E.D.C., Peruch L.A.M., Neubert E.D.O., Rocha M., Maraschin M. (2014). Metabolomics combined with chemometric tools (PCA, HCA, PLS-DA and SVM) for screening cassava (Manihot esculenta Crantz) roots during postharvest physiological deterioration. Food Chem..

[18] Mohd Ali M., Hashim N., Abd Aziz S., Lasekan O. (2022). Quality prediction of different pineapple (Ananas comosus) varieties during storage using infrared thermal imaging technique. Food Control.

[19] Ishimwe R., Abutaleb K., Ahmed F. (2014). Applications of thermal imaging in agriculture—a review. Adv. Rem. Sens..

[20] Zeng X., Miao Y., Ubaid S., Gao X., Zhuang S. (2020). Detection and classification of bruises of pears based on thermal images. Postharvest Biol. Technol..

[21] Jorge Aldave I., Venegas Bosom P., Vega González L., López De Santiago I., Vollheim B., Krausz L., Georges M. (2013). Review of thermal imaging systems in composite defect detection. Infrared Phys. Technol..

[22] Maldague X. (2001). Theory and practice of infrared technology for nondestructive testing.

[23] Djabou A.S.M., Carvalho L.J.C.B., Li Q.X., Niemenak N., Chen S. (2017). Cassava postharvest physiological deterioration: a complex phenomenon involving calcium signaling, reactive oxygen species and programmed cell death. Acta Physiol. Plant..

[24] Naik S., Patel B. (2017). 2017 International Conference on Emerging Trends & Innovation in ICT.

[25] Ding L., Dong D., Jiao L., Zheng W. (2017). Potential using of infrared thermal imaging to detect volatile compounds released from decayed grapes. PLoS One.

[26] Chandel A.K., Khot L.R., Osroosh Y., Peters T.R. (2018). Thermal-RGB imager derived in-field apple surface temperature estimates for sunburn management. Agric. For. Meteorol..

[27] Kuzy J., Jiang Y., Li C. (2018). Blueberry bruise detection by pulsed thermographic imaging. Postharvest Biol. Technol..

[28] Raza S.E.A., Prince G., Clarkson J.P., Rajpoot N.M. (2015). Automatic detection of diseased tomato plants using thermal and stereo visible light images. PLoS One.

[29] Rahman M.M., Barua P. (2021). A CNN model-based ensemble approach for fruit identification using seed, 2021 5th international conference on electrical information and communication technology. EICT.

[30] Akkaya B. (2019).

[31] Narayan Y. (2021). Hb vsEMG signal classification with time domain and Frequency domain features using LDA and ANN classifier. Mater Today Proc.

[32] Xiong T., Cherkassky V. (2005). A combined SVM and LDA approach for classification. Proceedings of the International Joint Conference on Neural Networks.

[33] Alajas O.J., Concepcion R., Dadios E., Sybingco E., Mendigoria C.H., Aquino H. (2021).

[34] Sucipto A., Tamrin T., Zyen A.K., Mulyo H. (2021).

[35] Karamizadeh S., Abdullah S.M., Halimi M., Shayan J., Rajabi M.J. (2014). Advantage and drawback of support vector machine functionality, I4CT 2014 - 1st international conference on computer, communications, and control technology. Proceedings.

[36] Shi L., Duan Q., Ma X., Weng M. (2012). The research of support vector machine in agricultural data classification. IFIP Adv. Inf. Commun. Technol..

[37] Binkhonain M., Zhao L. (2019). A review of machine learning algorithms for identification and classification of non-functional requirements. Expert Syst. Appl. X.

[38] Soofi A.A., Awan A. (2017). Classification techniques in machine learning: applications and issues. J. Basic Appl. Sci..

[39] Rajagopalan B., Lall U. (1999). A k-nearest-neighbor simulator for daily precipitation and other weather variables. Water Resour. Res..

[40] Waleed M., Um T.W., Kamal T., Usman S.M. (2021). Classification of agriculture farm machinery using machine learning and internet of things. Symmetry.

[41] Twa M.D., Parthasarathy S., Roberts C., Mahmoud A.M., Raasch T.W., Bullimore M.A. (2005). Automated decision tree classification of corneal shape. Optom. Vis. Sci..

[42] Luo Y., Shi H., Zhang Z., Zhang C., Zhou W., Pan G., Wang W. (2023). Wave field predictions using a multi-layer perceptron and decision tree model based on physical principles: a case study at the Pearl River Estuary. Ocean Engineering.

[43] Brodley C.E., Utgoo P.E. (1992).

[44] Ehsani S., Yazdanpanah H., Parastar H. (2023). Development of a non-targeted approach using three handheld spectrometers combined with ensemble classifiers for authentication of bovine milk. Chemometr. Intell. Lab. Syst..

[45] Hameed K., Chai D., Rassau A. (2020). A progressive weighted average weight optimisation ensemble technique for fruit and vegetable classification. 16th IEEE International Conference on Control, Automation, Robotics and Vision, ICARCV.

[46] Rahimikollu J., Das J. (2022). A supervised take on dimensionality reduction via hybrid subset selection. Patterns.

[47] Yogesh Y., Dubey A.K., Arora R.R. (2018). A comparative approach of segmentation methods using thermal images of apple, 2018 7th international conference on reliability, infocom technologies and optimization: trends and future directions. ICRITO.

[48] Xu H., Ying Y. (2004). Detecting citrus in a tree canopy using infrared thermal imaging. Monitoring Food Safety, Agriculture, and Plant Health.

[49] Melesse T.Y., Bollo M., Di Pasquale V., Centro F., Riemma S. (2022). Machine learning-based digital twin for monitoring fruit quality evolution. Procedia Comput. Sci..

[50] J.K. Afriyie, H. Rajakaruna, M.A.A. Nazha, F.K. Forson, Mathematical Modelling and Validation of the Drying Process in a Chimney-dependent Solar Crop Dryer, Energy Convers. (n.d.).

[51] Luna J., Dufour D., Tran T., Pizarro M., Calle F., García Domínguez M., Hurtado I.M., Sánchez T., Ceballos H. (2021). Post-harvest physiological deterioration in several cassava genotypes over sequential harvests and effect of pruning prior to harvest. Int. J. Food Sci. Technol..

[52] Masamba K., Changadeya W., Ntawuruhunga P., Pankomera P., Mbewe W., Chipungu F. (2022). Exploring farmers' knowledge and approaches for reducing post-harvest physiological deterioration of cassava roots in Malawi. Sustainability Assessment Journal.

[53] Uarrota V.G., Maraschin M. (2015). Metabolomic, enzymatic, and histochemical analyzes of cassava roots during postharvest physiological deterioration Bioinformatics. BMC Res. Notes.

[54] Baranowski P., Lipecki J., Mazurek W., Walczak R.T. (2008). Detection of watercore in ‘Gloster’ apples using thermography. Postharvest Biol. Technol..

[55] Van Zeebroeck M., Van linden V., Darius P., De Ketelaere B., Ramon H., Tijskens E. (2007). The effect of fruit factors on the bruise susceptibility of apples. Postharvest Biol. Technol..

